# Protozoan Parasites of Bivalve Molluscs: Literature Follows Culture

**DOI:** 10.1371/journal.pone.0100872

**Published:** 2014-06-23

**Authors:** José A. Fernández Robledo, Gerardo R. Vasta, Nicholas R. Record

**Affiliations:** 1 Bigelow Laboratory for Ocean Sciences, Boothbay, Maine, United States of America; 2 Department of Microbiology and Immunology, University of Maryland Baltimore, School of Medicine, Institute of Marine and Environmental Technology, Baltimore, Maryland, United States of America; Catalan Institute for Water Research (ICRA), Spain

## Abstract

Bivalve molluscs are key components of the estuarine environments as contributors to the trophic chain, and as filter –feeders, for maintaining ecosystem integrity. Further, clams, oysters, and scallops are commercially exploited around the world both as traditional local shellfisheries, and as intensive or semi–intensive farming systems. During the past decades, populations of those species deemed of environmental or commercial interest have been subject to close monitoring given the realization that these can suffer significant decline, sometimes irreversible, due to overharvesting, environmental pollution, or disease. Protozoans of the genera *Perkinsus*, *Haplosporidium*, *Marteilia*, and *Bonamia* are currently recognized as major threats for natural and farmed bivalve populations. Since their identification, however, the variable publication rates of research studies addressing these parasitic diseases do not always appear to reflect their highly significant environmental and economic impact. Here we analyzed the peer– reviewed literature since the initial description of these parasites with the goal of identifying potential milestone discoveries or achievements that may have driven the intensity of the research in subsequent years, and significantly increased publication rates. Our analysis revealed that after initial description of the parasite as the etiological agent of a given disease, there is a time lag before a maximal number of yearly publications are reached. This has already taken place for most of them and has been followed by a decrease in publication rates over the last decade (20– to 30– year lifetime in the literature). Autocorrelation analyses, however, suggested that advances in parasite purification and culture methodologies positively drive publication rates, most likely because they usually lead to novel molecular tools and resources, promoting mechanistic studies. Understanding these trends should help researchers in prioritizing research efforts for these and other protozoan parasites, together with their development as model systems for further basic and translational research in parasitic diseases.

## Introduction

Mollusc bivalves are key components of marine and estuarine environments because, as filter feeders, they play a critical role in maintaining water quality and ecosystem integrity. Marine bivalves are bottom dwellers attached to marine substrates for most of their life (*e.g.* oysters) or buried in the sand (*e.g.* clams, cockles); they can be displaced by heavy storms or moved as a result of human activities. The historical record indicates that bivalves were an abundant resource for the coastal inhabitants, although nowadays traditional local harvesting of the natural populations is being substituted worldwide by semi– intensive aquaculture initiatives. According to the Food and Agriculture Organization of the United Nations (http://www.fao.org/), the production of farmed clams, mussels, oysters, and scallops reached 14, 297, 010 metric tones with an estimated value of $13.7 bn (2010 data).

Protozoan parasites of the genera *Perkinsus*, *Haplosporidium*, *Marteilia*, and *Bonamia* severely affect a variety of mollusc species commercially harvested or farmed around the world. In particular, *Perkinsus marinus, Perkinsus olseni*, *Marteilia refringens*, *Bonamia ostreae*, and *Bonamia exitiosa* can infect abalones, clams, mussels, oyster, and scallops, and thus, are currently under surveillance by the World Organization for Animal Health (OIE; http://www.oie.int/; Aquatic Animal Health Code, Section 11: Diseases of Molluscs). Global warming and bivalve trading have been proposed as two of the main causes for expansion of parasitic diseases in molluscs [1]–[4]. As invertebrates lack adaptive immunity (although some immune memory has been proposed [5]), no vaccination approaches are feasible for bivalve molluscs, and intervention strategies are limited to management of the resource to enable commercialization of infected individuals prior to health decline or death, together with measures aimed at preventing the expansion of the parasite distribution range. Although selective breeding of individuals resistant to protozoan parasites has had variable success, it still remains as the most promising alternative [6]–[8]. Finally, the identification and use of anti– protozoan chemical agents has been hindered by the difficulties in treating marine molluscs due to both the inherent environmental toxicity of some of the compounds, and the farming practices in uncontained aquaculture settings [9]–[11]. Since their identification, however, the variable publication rates of research studies addressing the aforementioned parasitic diseases throughout the years do not always appear to reflect their highly significant environmental and economic impact. Previous quantitative analyses of trends in the literature have been used to investigate ecological hypothesis [12]. Here, we analyzed the peer– reviewed literature since the initial description of the aforementioned parasites, with the goal of identifying potential milestone discoveries or achievements that may have driven the intensity of the research in subsequent years, and significantly contributed to increase publication rates. Autocorrelation analyses suggested that advances in parasite purification and culture methodologies positively drive publication rates, most likely because they usually lead to novel molecular tools (*e.g.* transfection methodologies), resources (*e.g.* purification of proteins for drug profiling, crystallographic studies, antibody production), transcriptome and genome sequences, and ultimately, promote cellular and molecular mechanistic studies. These trends should help researchers and funding agencies in prioritizing research efforts for these and other protozoan parasites.

## Materials and Methods

A search of the SCOPUS database (http://www.info.sciverse.com/scopus/), which contains over 20, 500 titles from 5,000 publishers worldwide and it goes back to 1823, was carried out following Ward and Lafferty [12], for peer– reviewed articles published since 1950 until 2013 with titles or abstracts (abstracts were available for most articles) containing specific protozoan parasite taxonomic or disease name strings (Table 1). The starting date chosen corresponded with the first record of the mass mortality attributed to the corresponding pathogen under analysis, and descriptive and taxonomy studies were included [12], whereas references to meeting proceedings were excluded. The data for comparing the numbers of published articles for each parasite was restricted to the period from 1980 to 2013, since prior to 1980 the number of papers on the protozoan parasites was negligible. References were imported to EndNote (Thompson-Reuters) and titles and abstracts were manually curated by eliminating duplicities; the search returned no false positives with the exception of *Bonamia*, which shares its name with plants of the Convolvulaceae (*e.g. Bonamia spectabilis*). Once in EndNote the references were manually searched for each parasite and sorted by year, and the number of publications was then used to build an Excel spreadsheet to generate the plots. Manual curation was used to assign the work to a particular geographic area/country; when the publication involved authors from several countries, the country that originated the sample took priority over where the work was performed. The generated time series of numbers of papers were analyzed for statistically significant trends, autocorrelation, and dominant periodicities. To standardize the lengths of the time series, the autocorrelation was performed on the period 1980–2013, a period during which all parasites analyzed were under investigation. The analysis was repeated using the 30 years after first record for each parasite. To determine dominant periodicities, a spectral analysis on the de– trended time series was carried out using a Fourier transform [13], and peaks were identified in the power spectra.

**Table 1:** Search strings for protozoan parasites and bivalve groups.

	Search String
Parasite	
Bonamia	Bonamia, Bonamiosis, Microcytos
Haplosporidium	Haplosporidium, MSX (Multi- nucleated Sphere X), Minchinia
Marteilia	Marteilia, Marteiloides, Aber Disease
Perkinsus	Dermo, Dermocystidium, Labyrinthomyxa, Perkinsosis, Pseudoperkinsus
**Host** *	
Oysters	Crassostrea, Ostrea, oysters
Clams	Clams, Tapes, Ruditapes, Venerupis

*Abalones, mussels, cockles, and scallops, which can also be infected by some of these protozoan parasites, were not included in the host search for percent of literature reporting diseases.

## Results

**Figure 1:**
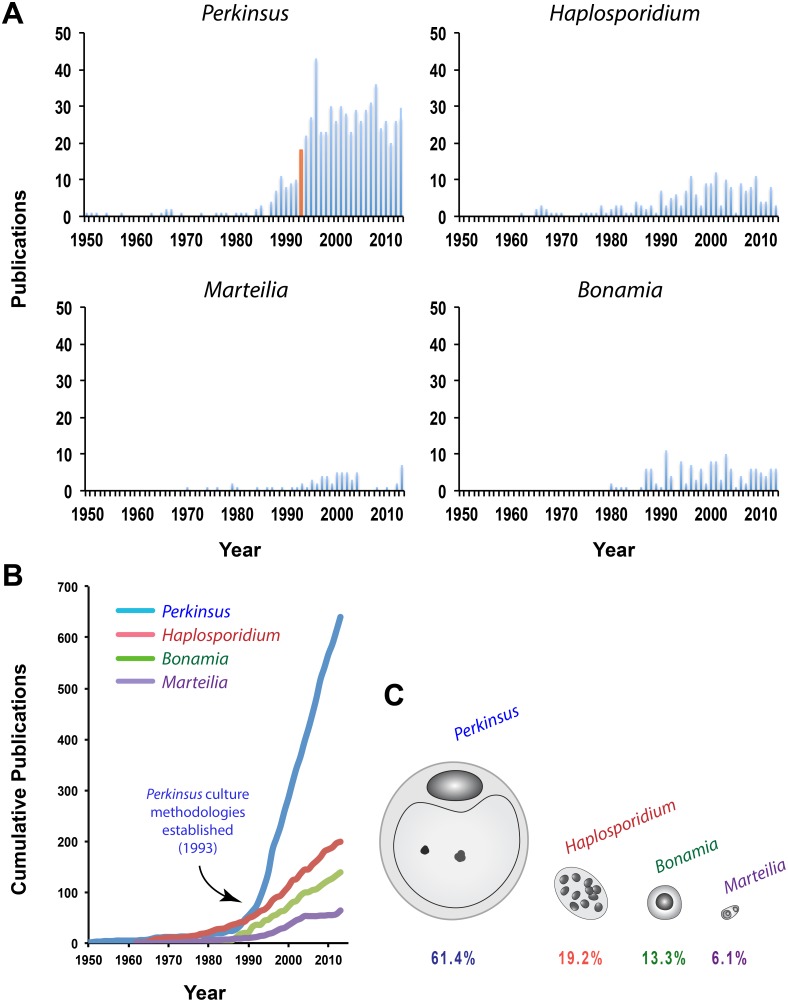
Number of papers in the literature (SCOPUS Database) reporting in the genera *Perkinsus*, *Haplosporidium*, *Marteilia*, and *Bonamia*; column in orange correspond to 1993, the year when the methodologies for culturing *Perkinsus* were published (A). Cumulative papers over the same period of time (B). Percent of the literature reporting each particular protozoan parasite (C).

**Figure 2:**
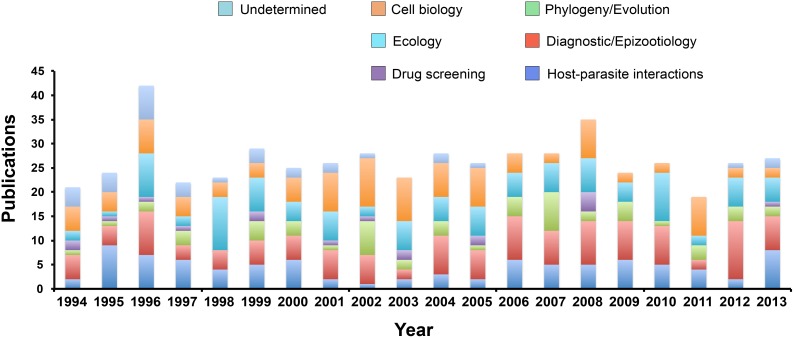
Number of papers in the literature (SCOPUS Database) reporting in the genera *Perkinsus*, *Haplosporidium*, *Marteilia*, and *Bonamia* distributed into each of the six categories; papers that did not fall into those categories were assigned to the undetermined category.

*Perkinsus marinus* was first described as associated with mass mortalities of eastern oysters in the Gulf coast during the 1950’s. Although the number of published papers was negligible during the following few years, during the late 1980’s and early 1990’s there was a sharp increase in publication rate, reaching more than 20 papers a year, a number that has remained relatively constant since then, and is significantly higher than those related to the other three parasite genera (*Haplosporidium*, *Marteilia*, and *Bonamia*) under study here (Figure 1A). For *Haplosporidium*, published articles appeared in the 1960’s, with most reports published in the 1990’s (though always below 10 papers/year) (Figure 1A). *Marteilia refringens* was first described in the 1970’s, with publications peaking in the 1990’s –early 2000’s, with a sharp decline over the last decade, and a spike in publications during 2013 (Figure 1A). The detection and description of *Bonamia ostreae* began in the early 1980’s and the profile of publication numbers, always lower than *Perkinsus* spp. and *Haplosporidium* spp., has been variable over the last three decades (Figure 1A). The number of spp. papers published on *Perkinsus* spp. has been about 5– to 13– fold over the other three protozoan parasites, and almost 3– fold over the three other species combined (Figure 1B). The autocorrelation analysis was carried out both on the four time series using data from 1980–2013, and using the first 30 years after the first record for each parasite. Using the 1980–2013 period, the *Perkinsus* spp. time series was significantly auto– correlated (p<0.05) out to a lag of nine years. *Haplosporidium* spp. and *Bonamia* spp. had no statistically significant autocorrelation, and *Marteilia* spp. had a significant two– year autocorrelation. All time series except *Marteilia* spp. had statistically significant increasing trends. For the de– trended data, *Perkinsus* spp. was significantly auto–correlated out to a lag of six years, and the other auto– correlations were unchanged. Using the 30 years following first record, results changed only for *Perkinsus* spp., which had no statistically significant auto– correlation or trend during this period (which predates the sharp increase in the 1990s), and for *Marteilia* spp., which was autocorrelated out to four years. In the spectral analysis, *Haplosporidium* spp. and *Marteilia* spp. had maximum peaks at a 20–30 year period and were mostly flat elsewhere, showing a strong 20–30 year mode underlying the signal. *Bonamia* spp. had a peak at 30 years as well, but also had comparable peaks at lower periods. *Perkinsus* spp. had a single maximum at 63 years, the full length of the time series, reflecting the dominance of the step change in the early 1990s. For the purpose of this study and based on the content, the papers were classified in six categories including cell biology, ecology, drug screening, phylogeny/evolution, diagnostic/epizootiology, and host– parasite interactions; papers that did not fall into these categories were assigned to the undetermined category (Figure 2).

**Figure 3:**
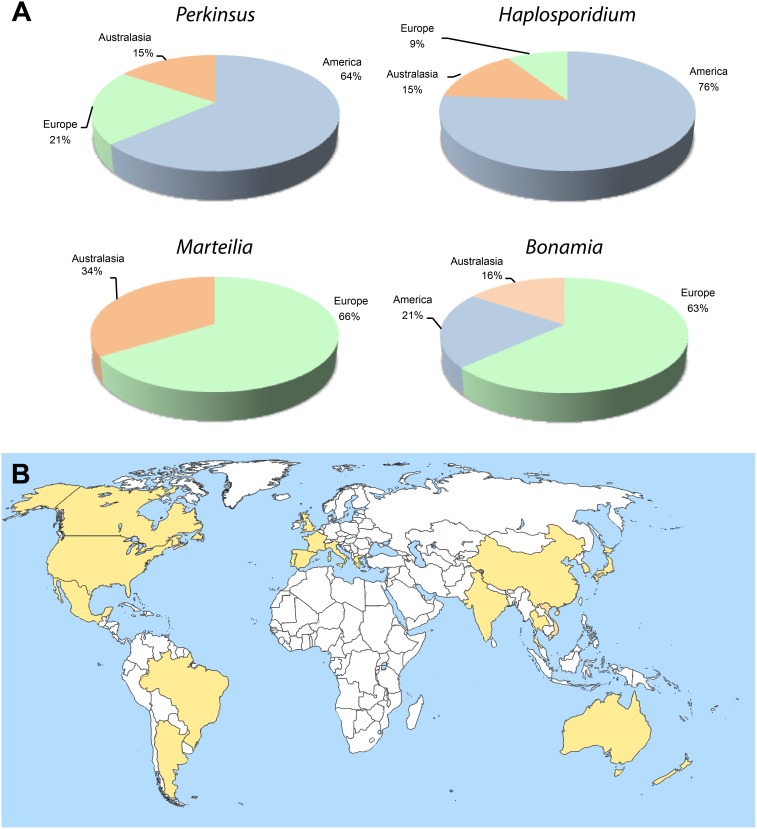
Distribution by continent of published papers in the literature reporting in the genera *Perkinsus*, *Haplosporidium*, *Marteilia*, and *Bonamia* (A). Countries where *Perkinsus* spp. have been reported by 2013 (B).

**Figure 4:**
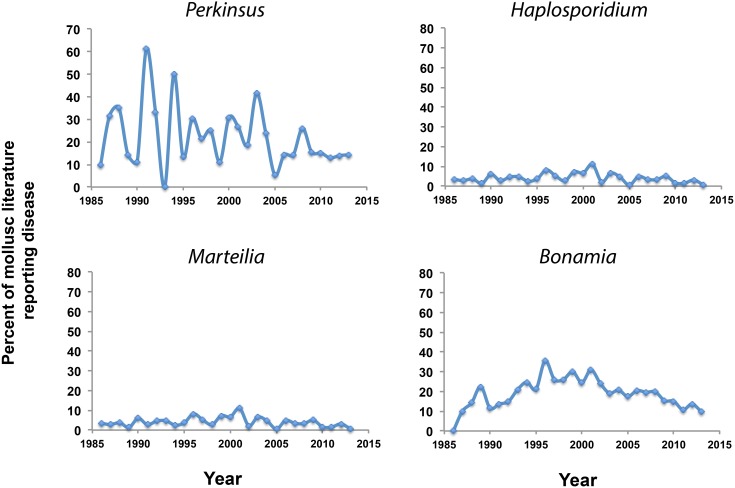
Percent of the literature (SCOPUS Database) in mollusc bivalves reporting disease by the protozoan parasites of the genera *Perkinsus*, *Haplosporidium*, *Marteilia*, and *Bonamia*.

Although *Perkinsus* spp. have been detected in 20 countries, making it the protozoan parasite of molluscs with the widest range distribution reported to date (Figure 3B), most papers (64%) on *Perkinsus* spp. correspond to North America (Figure 3A). In the case of *Haplosporidium* spp., it is the North American continent where more papers have been published (76%), followed by Australasia (Figure 3B). *Bonamia* and *Marteilia* papers were mostly published with reference to European countries (64% and 65%, respectively) although in the case of *Bonamia* spp., the number of published papers is also noticeable from both North America and Australasia (Figure 3A). Overall, the number of papers published on any particular protozoan parasite in relation to the papers published on their host has been decreasing during the last decades (Figure 4).

## Discussion

The protozoan parasites of the genera *Perkinsus*, *Haplosporidium*, *Marteilia*, and *Bonamia* have been described several decades ago but still affect significantly mollusc species that are environmentally and commercially relevant around the world, and are currently under surveillance by the OIE. Since no effective ant– parasitic treatments have been developed so far, the available intervention strategies are mostly limited to the improvement of management practices. Since their identification, however, the variable publication rates of research studies addressing these parasitic diseases do not always appear to reflect their highly significant environmental and economic impact. Here we analyzed the peer– reviewed literature since the initial description of these parasites with the goal of identifying potential milestone discoveries or achievements that may have driven the intensity of the research in subsequent years, and significantly increased publication rates. We did incorporate into this study protozoan parasites in the genus *Haplosporidium* [14], which, although they are not included in the OIE list of diseases, also have been associated to mass mortalities of oysters [15]. Although the herpes virus affecting Japanese oysters (*Crassostrea gigas*) has very recently become a problem especially for the production of spat [16], [17], this pathogen was not included in this study. This literature analysis initially confirmed that mass mortalities of bivalves were the main factor that prompted research aimed at the identification and description of the parasite as etiological agents. For example, although *Perkinsus marinus* (“Dermo” disease) was described upon mass mortalities of the eastern oyster, *Crassostrea virginica*, in Louisiana (USA) [18], [19], the analysis of archived material indicates that the parasite might have already been present in oysters from the same area *circa* 1920 [18]. In the late 1950’s, 90–95% of the oysters in Delaware Bay (USA) were killed by a disease named as MSX (Multinucleated Sphere X for “unknown”), the causative agent later identified as *Minchinia nelsoni* (currently *Haplosporidium nelsoni*) [15]. *Marteilia refringens* (“Aber” disease) was associated with mortalities of the European flat oyster, *Ostrea edulis*, in Aber Wrac’h, Brittany (France) in the 1970’s [20], [21]; similarly, *Bonamia ostreae* was also described after mass mortalities of *O. edulis* in Brittany (France) in 1979 [22]. There are several working hypotheses that could explain the onset of the epizooty, including the introduction of non– native host or parasite species, environmental conditions affecting the ability of the host to fight the parasites, and strains of variable virulence reaching the host (short generation time and large population size in the protozoan parasites provides the base for a high evolvability compared to the host). Testing this hypothesis, however, has proven to be not a trivial task since it would require rigorous examination of archived material that is not always available for the above– mentioned species. Thus we were interested in determining, once the etiological agent had been identified, how the studies on these diseases may have progressed over the last four to six decades. Remarkably, for the protozoan parasites analyzed here, there was a lag period between the description of the etiological agent and a noticeable increase in the number of published reports. Since their identification, the number of papers/year for all four parasites, have reached the maximum sometime during the past few years, and although the diseases they cause have not been resolved a significant decrease can be observed during the last decade. An exception is the noticeable rebound of published papers that has taken place for *Marteilia* in 2013; however, the next few years will reveal if this trend will continue. Interestingly, this overall decrease in the total of number of papers has been also accompanied by a decrease in the percent of the literature reporting the disease, reaching, in the last decade, the lowest ratios of papers on diseases compared to total papers on molluscs. This trend could be interpreted as a reduction of field monitoring programs, together with the acceptance that although the parasite is still a major concern and the management strategies available are judged sufficient [23]. The interested parties may put these to practice in the field without being reported in the peer– reviewed literature. Remarkably, and with the exception of Dermo and likely derived from the establishment of the culture methodologies, it appears that the numbers of papers published on these parasitic diseases follow 20– to 30– year modes. The local commercial importance of the shellfishery and the impact of the disease clearly play a significant role in the frequency of reports emerging from a particular area. Since the availability of research funds is a key factor that contributes to the publication rates, we attempted to identify those grants that specifically concern any aspects of the parasitic diseases addressed in this study. This search was limited to the National Science Foundation (NSF), because a searchable database for funded grant proposals is readily available (http://nsf.gov/awardsearch/). Since 1996, eighteen projects on *Perkinsus* biology (four of them include oyster resistance to both *Perkinsus* and *Haplosporidium*) have been funded by this agency, all of these addressing mechanistic or ecological aspects of Dermo disease. Nevertheless, it is also clear that programs supported by other funding agencies such as the state Sea Grant offices, and the Oyster Disease Research Program from the National Oceanic and Atmospheric Administration, have been and are still vital for supporting research on multiple aspects of these diseases in the USA, in both basic and translational studies. Studies on *Bonamia* spp. have been more prevalent in Europe where it depleted the flat oyster populations and still remains the main hindrance for the recovery of that shellfishery (http://oysterecover.eu/). Overall, the number of papers published on *Perkinsus* spp. greatly exceeds those published on the other three protozoan parasites combined and appears to have reached a plateau of paper/year far high than the other three parasite groups that are clearly beyond the plateau. This could be attributed not only to the widely recognized economic and environmental relevance of the eastern oyster in the coastal areas of USA and the significant detrimental impact of Dermo infections, but also to an increase in the number of *Perkinsus* spp. described worldwide [24]–[29], with first reports in Brazil, West coast of North America, China, Japan, and other regions in Asia [30]–[33]. The autocorrelation analysis suggested, however, that the major determinant in the increase of published papers was the establishment and optimization of the culture methodologies [34]–[37]. The strong autocorrelation in the *Perkinsus* time series implies that an increase in publications in one year corresponds to greater publications in subsequent years. The fact that this autocorrelation was absent in the time preceding the advances in culture methodologies for *Perkinsus*, as well as in the time series for the three other parasites, implies that these methodologies were the driving force in establishing momentum for the study of a parasite. Otherwise, the amount of research devoted to any particular parasite is effectively random from year to year, overlaid on a 20–30 year profile of rise and decline. Consequently, having unlimited amounts of the parasite infective stage has resulted in the development of diagnostic tools [38]–[42], essential to the description of new species [24]–[27], [29], more detailed epizootiology maps [2], [43], robust phylogenies [44], virulence studies [45]–[51], defense against the host responses [52]–[56], proteomics [57], and for the development of assays for the identification of drugs for intervention [9]–[11], [58]–[60]. Furthermore, it also accelerated the understanding of *Perkinsus*’ biology, including the parasite’s mechanisms for entry, survival, proliferation inside the host [53]–[56], [61]–[73], and factors controlling zoosporulation [74], as well as biotechnology and biomedicine applications [70], [75]. Moreover, the availability of the transcriptome [76], and the genome (http://www.ncbi.nlm.nih.gov/nuccore/126302507), together with recently developed transfection methodology [77], is likely to result in making *Perkinsus* a model organism to study protozoan parasitic diseases [78]. Over time, the most represented categories of papers included diagnostic/epizootiology, host – parasite interactions, and cell biology. The large numbers of diagnostic/epizootiology studies can be attributed to the increasing number of countries performing surveys as a result of current concerns about Dermo disease associated to the increase in worldwide trade of bivalve species. The increase in publications on host – parasite interactions and cell biology of the parasite suggests that more research groups are focusing on the mechanistic aspects of the parasitic disease. At a lesser scale, the development of methods for purification of *Bonamia* cells [79], also has favored several mechanistic studies [80]–[82]. Similarly, the identification of a probable intermediate host in the life cycle for *Marteilia refringens* [83], [84], appears to be responsible for the noticeable increase in publications during 2013.

**Figure 5:**
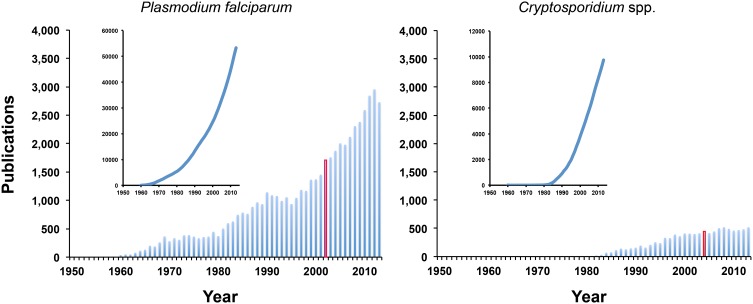
Number of papers published every year (1960–2013) and cumulative papers (inset figures) in the literature (SCOPUS Database) using *Plasmodium* and *Cryptosporidium* as search strings. Columns in red correspond to the years when the genomes were published.

It would be expected that the sequencing of the genomes of protozoan parasites (as well as those of their hosts) would significantly increase not only the publication rates, but also the number of mechanistic studies. Surprisingly, the publication of the genomes of *Plasmodium falciparum* and *Cryptosporidium* spp. [85]–[87]) did not result in the expected increase in the total number of papers published per year (Figure 5); instead, both *Plasmodium* and *Cryptosporidium* species are highly autocorrelated out to >20 years, indicating that the amount of papers in any given year is strongly connected to the number of papers in previous years. Both time series have an underlying periodicity of 20–30 years, though for *P. falciparum*, the dominant period is the full length of the time series. This trend is perhaps the result of the continued high publication rates for these two particular parasite species during the past decade (in the range of 2,300 papers/year for *Plasmodium* and 474 papers/year for *Cryptosporidium*) and the fact that the full length sequences for numerous genes had been available much earlier than their genome drafts. It remains to be seen in the next few years if the availability of genome sequences for parasites from bivalve molluscs, obviously less popular than *Plasmodium* and *Cryptosporidium*, will significantly change the publication rates, particularly on mechanistic studies that may lead to the identification of therapeutic or ecological targets and the development of new intervention strategies against the disease to complement available strategies [23]. Finally, this is the first study that provides a side– by– side comparison of the publication record for the four main genera of protozoan parasites affecting molluscs of commercial interest. The trends identified here should help researchers in the field to fine– tune their research projects during these challenging times when available funding for research is limited, and to students interested in pursuing career in marine parasitology to make a more educated decision looking into their future careers. State and local agencies can also benefit from this study by prioritizing or compartmentalizing research efforts for these and other protozoan parasites, especially when the initial funding effort derived from a catastrophic event (such as mass mortalities) starts fading. Under these circumstances, the continuity of the research projects on a particular protozoan parasite depends on available resources and tools that are required to make it competitive with other model systems.
